# Trace Amine-Associated Receptor 2 Is Expressed in the Limbic Brain Areas and Is Involved in Dopamine Regulation and Adult Neurogenesis

**DOI:** 10.3389/fnbeh.2022.847410

**Published:** 2022-04-01

**Authors:** Evgeniya V. Efimova, Saveliy R. Kuvarzin, Mikael S. Mor, Nataliia V. Katolikova, Taisiia S. Shemiakova, Valeria Razenkova, Maria Ptukha, Alena A. Kozlova, Ramilya Z. Murtazina, Daria Smirnova, Aleksandr A. Veshchitskii, Natalia S. Merkulyeva, Anna B. Volnova, Pavel E. Musienko, Dmitrii E. Korzhevskii, Evgeny A. Budygin, Raul R. Gainetdinov

**Affiliations:** ^1^Institute of Translational Biomedicine, St. Petersburg State University, St. Petersburg, Russia; ^2^Institute of Experimental Medicine, St. Petersburg, Russia; ^3^Pavlov Institute of Physiology Russian Academy of Sciences, St. Petersburg, Russia; ^4^Department of Neurobiology, Sirius University of Science and Technology, Sochi, Russia; ^5^St. Petersburg University Hospital, St. Petersburg State University, St. Petersburg, Russia

**Keywords:** trace amines, trace amine-associated receptors (TAARs), TAAR2, limbic system, dopamine, BDNF, neurogenesis

## Abstract

Trace amines are a group of biogenic amines that are structurally and functionally close to classical monoamine neurotransmitters. Trace amine-associated receptors (TAARs) are emerging as promising targets for treating neuropsychiatric disorders. It has been documented that all TAARs, apart from TAAR1, function as olfactory receptors involved in sensing innate odors encoded by volatile amines. However, recently, brain expression and function of TAAR5 were also demonstrated. In this study, we assessed the behavior, brain neurochemistry, and electrophysiology changes in knock-out mice lacking Trace amine-associated receptor 2 (TAAR2) but expressing beta-Galactosidase mapping expression of TAAR2 receptors. As expected, we detected beta-Galactosidase staining in the glomerular layer of the olfactory bulb. However, we also found staining in the deeper layers of the olfactory bulb and several brain regions, including the hippocampus, cerebellum, cortex, raphe nuclei, hypothalamus, and habenula, indicating that TAAR2 receptors are not only expressed in the olfactory system but are also present in the limbic brain areas that receive olfactory input. In behavioral experiments, TAAR2 knock-out (TAAR2-KO) mice showed increased locomotor activity and less immobility in the forced swim test, with no changes in anxiety level. Furthermore, TAAR2-KO mice showed alterations in brain electrophysiological activity—particularly, decreased spectral power of the cortex and striatum in the 0, 9–20 Hz range. TAAR2-KO mice also had elevated tissue dopamine levels in the striatum and an increased dopaminergic neuron number in the Substantia Nigra. In addition, an increased brain-derived neurotrophic factor (BDNF) mRNA level in the striatum and Monoamine Oxidase B (MAO-B) mRNA level in the striatum and midbrain was found in TAAR2-KO mice. Importantly, TAAR2-KO mice demonstrated an increased neuroblast-like and proliferating cell number in the subventricular and subgranular zone, indicating increased adult neurogenesis. These data indicate that in addition to its role in the innate olfaction of volatile amines, TAAR2 is expressed in limbic brain areas and regulates the brain dopamine system, neuronal electrophysiological activity, and adult neurogenesis. These findings further corroborated observations in TAAR1-KO and TAAR5-KO mice, indicating common for TAAR family pattern of expression in limbic brain areas and role in regulating monoamine levels and adult neurogenesis, but with variable involvement of each subtype of TAAR receptors in these functions.

## Introduction

Trace amines are a group of biogenic amines that can be found endogenously and are represented mostly by derivatives of amino acids (Boulton, 1974). Trace amines and their involvement in neuropsychiatric disorders have been investigated for decades. However, they were generally recognized as just byproducts of amino acid metabolism without clear neurotransmitter function. In addition, their mechanism of action, beyond indirect modulation of the classical monoaminergic systems, remained unknown (Jones, 1983; Wolf and Mosnaim, 1983; Berry, 2004). The discovery of a family of G protein-coupled trace amine-associated receptors (TAARs) in 2001 indicated that trace amines might represent a novel neurotransmitter system (Borowsky et al., 2001; Bunzow et al., 2001). There is a growing interest in TAAR receptors as prospective targets for the therapy of neuropsychiatric disorders (Branchek and Blackburn, 2003; Lindemann and Hoener, 2005; Sotnikova et al., 2009; Rutigliano and Zucchi, 2020). Mammals have nine subtypes of TAAR receptors, and six of them are functional in humans (Lindemann and Hoener, 2005; Berry et al., 2017; Gainetdinov et al., 2018). TAAR1 receptor was shown to be expressed in the brain and became a significant point of interest for pharmacologists (Berry et al., 2017; Gainetdinov et al., 2018; Koblan et al., 2020). In contrast, all the other TAARs were mostly described as olfactory receptors that sense innate odors encoded by volatile amines (Liberles and Buck, 2006; Pacifico et al., 2012; Dewan, 2021). However, recent studies showed that the TAAR5 is also present in several brain areas, with the most prominent expression in the limbic structures receiving olfactory input (Espinoza et al., 2020; Efimova et al., 2021; Kalinina et al., 2021). TAAR5 knockout (TAAR5-KO) mice showed alterations in emotional behavior, decreased anxiety, and depression-like behavior. On the neurochemical level, TAAR5 and TAAR1 influenced the dopamine and serotonin systems. Furthermore, TAAR5-KO mice demonstrated increased striatal dopamine levels with the elevated number of dopaminergic neurons in the Substantia Nigra (SN) and adult neurogenesis markers (Espinoza et al., 2020; Efimova et al., 2021, 2022).

Another member of the TAAR receptor family, TAAR2, is phylogenetically close to the TAAR1 receptor (Lindemann and Hoener, 2005; Gainetdinov et al., 2018). As previously with TAAR5, only the olfactory function of TAAR2 was firmly established (Liberles and Buck, 2006; Pacifico et al., 2012; Dewan, 2021). However, several studies demonstrated its expression outside of olfactory epithelium, i.e., in leukocytes, gastrointestinal system, heart, lungs, and testis (Ito et al., 2009; Babusyte et al., 2013; Berry et al., 2017; Gainetdinov et al., 2018). Only limited information is available on the presence of TAAR2 in the brain, but an altered expression of TAAR2 mRNA was found in the hippocampus of the strain of rats with altered emotional status following incentive loss (Sabariego et al., 2013). No volatile amine or endogenous TAAR2 ligands have been identified so far. However, the structure of TAAR2 predicts that it should have a high affinity to primary amines (Ferrero et al., 2012; Cichero and Tonelli, 2017). The lack of selective ligands of TAAR2 made it challenging to determine its functions. In this study, we used TAAR2-KO mice with beta-Galactosidase (LacZ) insertion to explore the pattern of TAAR2 expression in the brain and determine its functions in brain regulation and behavior.

## Materials and Methods

### Generation of Trace Amine-Associated Receptor 2 Knock-Out Mice

**FIGURE 1:**
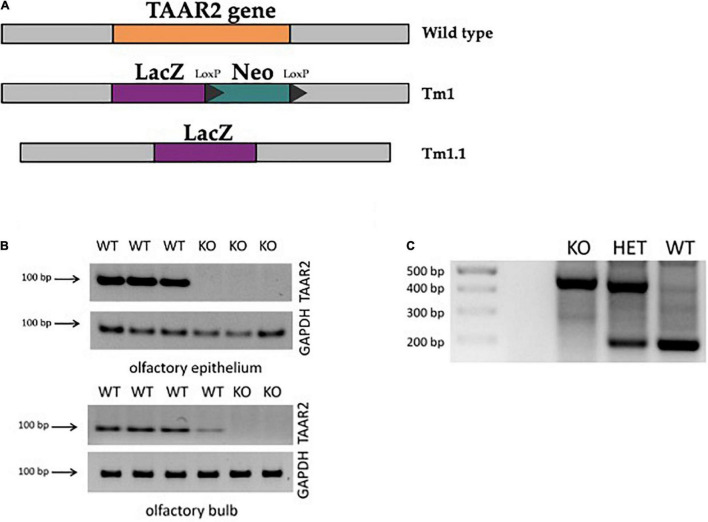
∣ Generation of trace amine-associated receptor 2 knockout (TAAR2-KO) mice. **(A)** TAAR2-KO mice were generated using homologous recombination with ZEN-Ub1 vector, which resulted in the deletion of the TAAR2 gene (Gene ID: 209512, MGI:2685071), and insertion of expression-selection cassette that contained LacZ (beta-galactosidase cording sequence from Escherichia *coli* LacZ gene) and Neo (cording sequence of neomycin) (Tm1) following Cre-mediated excision which resulted in the removal of the neomycin selection cassette leaving the inserted lacZ reporter sequence (Tm1.1). **(B)** The knockout of the TAAR2 gene was further confirmed by RT-PCR, in which the presence of TAAR2 mRNA was detected in the olfactory epithelium and olfactory bulb of the WT mice, but not in TAAR2 knockout mice. **(C)** Standard genotyping of TAAR2 knockout mice: genomic DNA was obtained from animal skin tissue of WT, TAAR2-HET, and TAAR2-KO mice. Knockout gene showed 400 bp band, wild-type gene showed 200 bp band.

The TAAR2-KO mouse strain [C57BL/6N *Taar2^TM1^.^1(KOMP)Vlcg^*/MbpMmucd, RRID: MMRRC_049963-UCD] was obtained from the Mutant Mouse Resource and Research Center (MMRRC) at the University of California at Davis, an NIH-funded strain repository. This mouse line was donated to the MMRRC by The Knockout Mouse Project (KOMP) Repository, University of California, Davis, which originated from Kent Lloyd, UC Davis Mouse Biology Program. This mouse strain was developed by methods previously described (Hall et al., 2009). Briefly, C57Bl/6N ES cell clone 11384A-E5 was injected into morulae or blastocysts. The resulting chimeras were mated to C57BL/6N mice, and heterozygous tm1 (Deletion) mice were obtained and bred to a ubiquitous Cre deleter mouse line for recombination of the LoxP sites to develop the tm1.1 (CREed Deletion) allele mice. Thus, the neomycin resistance gene selection cassette was removed in TAAR2-KO mice through CREed deletion (Figure 1A).

To further validate knockout, TAAR2 gene expression analysis by qPCR was performed. Tissues were dissected on ice, frozen in liquid nitrogen, and stored at −80°C. RNA isolation was performed using TRI Reagent (MRC, United States) according to the manufacturer’s instructions. To eliminate any remaining genomic DNA, TURBO DNA-free kit (Thermo Scientific, United States) was used on RNA samples. Genomic DNA contamination of total RNA was controlled using real time (RT) minus control. Reverse transcription and qPCR were performed as previously described (Kalinina et al., 2021). Briefly, 1 μg of RNA was taken for the synthesis of cDNA using Revertaid Reverse Transcriptase (Thermo Scientific, United States), and 1 μl of cDNA was used for qPCR. Primers for detection of mouse TAAR2 (mTAAR2_F: 5′-CGGATTCACCATCATGCCAT-3′, mTAAR2_R: 5′-CTAAG CATCAGGTCGAAGCT-3′) were tested for specific target amplification in the sample compared to the RT, which was controlled using melting curve analysis (from 55 to 95°C) and 2% agarose gel electrophoresis (Liberles and Buck, 2006). Mouse glyceraldehyde 3-phosphate dehydrogenase (GAPDH) gene (primers: mGAPDH_F2: 5′-ttgatggcaacaatctccac-3′, mGAPDH_R2: 5′-cgtcccgtagacaaaatggt-3′), a housekeeping gene, was included as an internal control. Implementing PCR with cDNA derived from the mouse olfactory epithelium and olfactory bulb, which constitutively express TAAR2, showed a prominent signal in wild-type (WT), but not TAAR2-KO mice (Figure 1B).

Before experiments, all mice were genotyped (Figure 1C). For genotyping, DNA was isolated from mice ear skin 1 mm × 1 mm fragment by overnight incubation in a lysis buffer [0.1 M Tris–HCl, 0.005 M ethylenediaminetetraacetic acid (EDTA), 0.2% sodium dodecyl sulfate, 10 mg/ml proteinase K] at +55°C. Proteinase K was then deactivated by heating the sample at +95°C, followed by adding 0.02 mg/ml Rnase A in Tris–EDTA buffer (1 M Tris–HCl, 0.1 M EDTA, pH 7.4). Relying on the insertion of the LacZ gene into the TAAR2 mice line, PCR was based on four primers: TAAR2-WT-F—CAAACAGCTAAGCACCACTACCACC (Reg-Taar2-wtF); TAAR2-WT-R—AATGGAGTCTGTGACAGCATTGTGC (Reg-Taar2-wtR); SU—ATCAGAATTGAGAGCCTCCG; and LacinZRev—GTCTGTCCTAGCTTCCTCACTG. This mixture was used to detect 200 or 400 bp length fragments or both, which related to TAAR2-WT, TAAR2-KO, and TAAR2-HET genotypes, respectively (Figure 1C). The PCR-performing program was conducted as follows: 95°C for 3:00 min; 10 cycles 95°C for 15 s, 65°C for 30 s—1°C per cycle, 72°C for 40 s; 29 cycles, 95°C for 15 s, 55°C for 30 s, 72°C for 40 s; then 72°C for 5 min. The reaction volume was 25 ul and contained 1 ul for each pair of primers (F/R—SU/LacZinRev and F/R—WT; 10 mM), BiolabMix x2—12.5 ul, MQ water—9.5 ul, and isolated DNA itself—1 ul. PCR products were run on a 2% agarose gel stained with 0.2 mg/ml of ethidium bromide.

### Animals

All procedures involving animals and their care were carried out following the guidelines established by the European Community Council (Directive 2010/63/EU of September 22, 2010) and were approved by the Ethics Committee of St. Petersburg State University, St. Petersburg, Russia. All experiments were performed in 3–6-month-old male TAAR2-KO and littermate WT mice. The mice were housed three to four per cage and maintained under standard lab conditions (12 h light/dark cycle, 21 ± 1°C, and 40–70% humidity) with food and water provided *ad libitum*.

### Trace Amine-Associated Receptor 2 LacZ Histochemistry

The analysis was performed as described (Espinoza et al., 2020). The mice (*n* = 7 for WT mice and *n* = 10 for TAAR2-KO group) were anesthetized with a mixture of 200 mg/kg zoletil and 16 mg/kg xylazine. Next, mice were transcardially perfused with 0.9% NaCl in 0.1 M phosphate-buffered saline (PBS) at pH = 7.4 followed by the 4% paraformaldehyde in 0.1 M PBS at pH 7.4. After perfusion, brains were cryoprotected in 20% and 30% sucrose solutions. Then, 50 μm frontal free-floating sections were prepared on a cryotome (Reichert, Vienna, Austria).

To identify beta-Galactosidase expression, LacZ staining standard protocol was performed. Before staining, tissue sections were washed 3 times for 10 min in 0.01 M PBS and incubated for 21 h in LacZ staining solution [1 mg/ml 5-bromo-4-chloro-3-indolyl-D-galactopyranoside, 5 mM K3Fe(CN)6, 5 mM K4Fe(CN)6, and 2 mM MgCl2 in PBS] at 37°C. The staining was stopped by washing the tissue sections 3 times for 10 min at room temperature in 0.01 M PBS. After the histochemical reaction, the sections were mounted at the glasses and coverslipped in Mowiol. The tissue sections were analyzed on an Olympus CX31 microscope (Olympus Corporation, Tokyo, Japan) with a built-in Nikon camera (D3200, Nikon Corporation, Tokyo, Japan).

### Behavioral Testing

The experiments were carried out in the following order: marble burying, light-dark transition test, open field, elevated plus maze, forced swim test, and hot plate test. Ten WT and 11 TAAR2-KO mice were used in each behavioral experiment. Fifteen WT and 21 TAAR2-KO mice were used for the locomotor activity test. All experiments were conducted during the light phase, and the same types of experiments were performed at the same short time period of the day.

#### Open Field Test, 5 min

The circular open field test was used to analyze the exploratory activity of mutant and control mice. The apparatus consisted of a gray plastic round arena (diameter, 63 cm) with 13 holes in the arena floor (hole diameter, 1.6 cm) and lighting conditions at 200 lx. Each mouse was placed at the center of the arena, and spontaneous exploration activity was recorded with the Noldus Ethovision video-tracking software for 5 min. The following behavioral parameters were monitored: total distance moved, velocity, cumulative duration in the central zone and frequency entering the central zone, total time of grooming, number of rearings, and number of holes explorations.

#### Locomotor Activity, 120 min

The horizontal locomotor activity of the mice was evaluated using Noldus Ethovision video-tracking software. The test was performed using a square open field apparatus that was novel to the animals. Mice were placed in plexiglass boxes 40 cm (L) × 40 cm (W) × 40 cm (H), and their horizontal activity was recorded for 120 min. No bedding, food, or water was provided to animals in this test.

#### Elevated Plus Maze Test

For measurement of the level of anxiety-like behavior, we performed the elevated plus-maze test. The maze consisted of two opposite open (30 × 5 cm) and two opposite enclosed arms (30 × 5 × 15 cm) elevated 40 cm from the floor. Mice were placed at the center of the elevated plus-maze facing the open arm and were recorded for 5 min. The following parameters were analyzed using the Noldus Ethovision software: the cumulative duration in open arms, the frequency of entrance in open arms, total distance moved separately in opened and closed arms, mean velocity, the number of rearing in closed arms, and the number of head dipping on open arms.

#### Forced Swim Test

The forced swim test was used to evaluate depressive-like behavior. Each mouse was individually put into a plexiglass cylinder (diameter: 10 cm, height: 21 cm) filled 2/3 of the way with water. The temperature of water was 24°C ± 1°C. Mice were forced to swim for 10 min. The water level was deep enough (18 cm) so the mouse’s tail did not touch the bottom. After the swim, mice were removed from the water, toweled dry, returned to their home cage, and made to remain in a warm environment before drying. The total time of immobilization and active and passive swimming was assessed.

#### Social Interaction Test

The social interaction test was used to quantitatively evaluate social behavior in mice shown toward a standard tester, i.e., a group-housed unfamiliar subject of the same strain, age, weight, and sex. The experimental apparatus was a plexiglass box [35 cm (L) × 48 cm (W) × 19 cm (H)] divided by two parts with a white baffle with a small passage, enough for a mouse to move freely between two parts of the apparatus. In each part, a small perforated cage [11 cm (L) × 10 cm (W) × 10 cm (H)] was placed. Mice were pre-habituated to the apparatus with two empty cages for 5 min. The next day, an unfamiliar same-sex mouse was placed into one of the wired cages. For tested mice, time in the zone near cages with or without mice and time of exploration of cages were registered. The zone near the cage was marked at 8 cm from the cage. Exploration of the cage was manually registered. Mice were considered to explore the cage if they performed sniffing and rearing to the cage.

#### Light-Dark Box Test

The apparatus consisted of two chambers [20 cm (L) × 20 cm (W) × 20 cm (H) each]. One of the chambers was highly illuminated, while another one remained dark. The chambers were connected with a passage that allowed mice to move freely between chambers. Each mouse was placed in the light part of the apparatus heading away from the dark chamber and was allowed to explore the apparatus for 3 min. The latency time to enter the dark chamber and the latency time to reenter the light chamber were recorded, along with the total time in the light chamber and the number of entrances to the light chamber.

#### Marble Burying

Each mouse was placed for 30 min in an opened plexiglass cage [35 cm (L) × 19 cm (W) × 14 cm (H)] filled with 5 cm of fresh wooden bedding with 5 rows of 3 marbles. The number of buried marbles were counted after 30 min of the experiment. Marbles were scored as buried if at least two-thirds of their surface area was covered by bedding.

#### Hot Plate Test

The hot plate test was conducted to measure the pain sensitivity. The test was done using the Bioseb BIO-CHP apparatus. Animals were placed on a cylindrical container, sized 350 × 170 × 170 mm, which was installed on a hot metal plate, sized 165 × 165 mm, which was maintained at 55°C. The elapsed time between the placement of the animal on the hot plate and the occurrence of the licking of the hind paws, shaking, or jumping off from the surface was recorded as response latency in seconds. Mice were removed if any kind of reaction to heat was displayed or after 30 s.

### Electrophysiological Recordings

#### Operating Procedure

Electrophysiological studies were conducted on 14 adult male mice animals: TAAR2-KO (*n* = 7) and WT (*n* = 7). Epidural screws were used for electrocorticogram (ECoG) recordings (0.5 mm in diameter; 1 mm in length; steel), while intracerebral electrodes were used for local field potential (LFP) recordings (50 μm in diameter; 1.2 mm/3.2 mm in length; tungsten wire in perfluoroalkoxy polymer isolation). For each animal, four electrodes were implanted under general anesthesia (200 mg/kg Zoletil intraperitoneally; Xylazine 0.2 mg/kg intramuscularly): epidural reference electrode (2 mm anterior to bregma and 1 mm lateral to midline); primary (bordering on secondary) motor cortex epidural electrode (1 mm anterior to bregma and 1 mm lateral to midline); primary somatosensory cortex intracerebral electrode (1.2 mm in length, 1.5 mm posterior to bregma and 2.5 mm lateral to midline); and striatal intracerebral electrode (3.2 mm in length, 0.5 mm posterior to bregma and 2.5 mm lateral to midline). Correct electrode placement was achieved by using a micromanipulator in a stereotaxic frame, in which the animal’s head was fixed during the whole operating procedure. Electrodes were fixed on the skull with dental cement.

#### Electrocorticogram and Local Field Potential Recordings

Experimental setting for electrophysiological recordings consisted of an amplifier (x1000 gain), analog-to-digital converter L-791 (L-card), and Bioactivity Recorder v5.44 software (Dmitrii A. Sibarov). During the recording process, the animal was placed in a 20 × 20 × 25 cm plexiglas box which, along with the amplifier, was located in an isolated grounded setting.

The brain activity of TAAR2-KO and WT groups was recorded for 20 min in awake, freely moving mice, 3 times for each animal on different days. Signal was band-pass filtered between 0.1 and 200 Hz and digitized with 2,500 samples per second per channel. Only those epochs in which mice exhibited similar behavior were included in the analysis. These recordings were then used to compare spectral characteristics of electrophysiological activity of TAAR2-KO and control mice.

#### Data Analysis

Using the Bioactivity Recorder software, 12 epochs without artifacts that were 20 s in length were picked out from each recording. Then Fourier transform was performed using Clampfit 10.2.0.16 software (MDS Analytical Technologies, United States). The resulting power spectra (frequency in Hz, power spectral density in μV^2^) of WT mice were compared to those of TAAR2-KO mutants. The data was analyzed after normalization, which transforms all values from one data set into percentages of the sum of all power spectra values. The resulting data was in the 0.9–20 Hz range after data in the 0–0.9 range was excluded due to the abundance of artifacts. Data were compared within specific ranges corresponding to the following electroencephalographic rhythms: delta (0.9–4 Hz), theta (4.5–9 Hz), alpha (9.5–12 Hz), and beta (12.5–20 Hz).

### High-Performance Liquid Chromatography Measurements of the Tissue Monoamine and Metabolites Content

High-performance liquid chromatography (HPLC) measurements of tissue serotonin (5-HT), dopamine (DA), norepinephrine (NE) and their metabolites 5-hydroxyin- doleacetic acid (5-HIAA), 3,4-Dihydroxyphenylacetic acid (DOPAC), and Homovanillic acid (HVA) were performed as described previously (Belov et al., 2019). Briefly, the striatum, the frontal cortex, the hypothalamus, and the hippocampus were dissected on ice, frozen in liquid nitrogen, and stored at −80°C. The samples for analysis were homogenized in 0.1 M HClO4, centrifuged (10 min, + 4°C; 14,000 × *g*), and filtered using centrifuge filter units [polyvinylidene fluoride (PVDF) membrane; pore size, 0.22 μm, Millipore, Burlington, MA, United States]. Measurements of monoamines in the tissue samples were performed using HPLC with electrochemical detection (Eicom, HTEC-500, Japan) with a carbon electrode WE-3G (Eicom, Japan) using +650 mV applied potential. The system was equipped with a reverse-phase column CA-50DS (150 × 2.1 mm, Eicom, Japan) at 200 μl/min flow rate. The mobile phase contained 100 mM sodium-phosphate buffer, 0.17 mM EDTA, 1.8 mM octanesulfonic acid sodium salt, and 18% (vol/vol) methanol, pH 4.5. All peaks obtained were normalized to internal standard 3,4-dihydroxybenzylamine, and final values for monoamines and metabolites levels were expressed as nanogram per milligram of wet tissue weight.

### Quantitative Real-Time PCR of Key Neuronal Markers

Mice (*n* = 6 for each group) were euthanized using cervical dislocation. Cortex, striatum, and midbrain with medulla oblongata were dissected on ice. Total RNA from each part was isolated using Trizol reagent (cat. # 15596018, Thermo Fisher Scientific, Waltham, MA, United States). cDNA was synthesized from 1 mkg of RNA using a RevertAid Reverse Transcriptase (cat. # EP0442, Thermo Fisher Scientific, Waltham, MA, United States). Quantitative PCRs were carried out with qPCR mix-HS SYBR (cat. # PK147L, Eurogen). The data were assessed using a delta-Ct method and normalized by the GAPDH expression. The used primer sequences were shown in Table 1.

**TABLE 1:** ∣ Primer sequences for RT-PCR.

	Forward	Reverse
GAPDH	TCAATGAAGGGGTCGTTGAT	CGTCCCGTAGACAAAATGGT
BDNF	TCATACTTCGGTTGCATGAAGG	AGACCTCTCGAACCTGCCC
MAO-B	ATGAGCAACAAAAGCGATGTGA	TCCTAATTGTGTAAGTCCTGCCT
TH	CGCCGTCCAATGAACCTT	CACTATGCCCACCCCCAG
DAT	GATGCACATAGCAGCAACTCT	GCACACCACGCTCAAAATACTC
COMT	CTGGGGTTGGTGGCTATTG	CCCACTCCTTCTCTGAGCAG
MAO-A	GCCCAGTATCACAGGCCAC	CGGGCTTCCAGAACCAAGA
D1R	CACGGCATCCATCCTTAACCT	TGCCTTCGGAGTCATCTTCCT
D2R long	AACTGTACCCACCCTGAGGA	GTTGCTATGTAGACCGTG
D2R short	CACCACTCAAGGATGCTGCCCG	GTTGCTATGTAGACCGTG
TPH2	GTGACCCTGAATCCGCCTG	GGTGCCGTACATGAGGACT
GAT1	GAAAGCTGTCTGATTCTGAGGTG	AGCAAACGATGATGGAGTCCC
NMDAR1	ATGCACCTGCTGACATTCG	TATTGGCCTGGTTTACTGCCT
AChE	CTCCCTGGTATCCCCTGCATA	GGATGCCCAGAAAAGCTGAGA
CDNF	AAGAAAACCGCCTGTGCTATT	TCTTCACGGCAGGTATGTGTA
GDNF	TCTTTCGATATTGCAGCGGTT	GTCACTTGTTAGCCTTCTACTC

### Tyrosine Hydroxylase Immunohistochemistry

Mice (*n* = 4 for each group) were transcardially perfused with 0.9% NaCl in 0.1 M PBS at pH 7.4 followed by the 4% paraformaldehyde in 0.1 M PBS at pH 7.4. Manipulations were performed under deep anesthesia with a mixture of 200 mg/kg zoletil and 16 mg/kg xylazine. After perfusion, the brain was removed and sequentially immersed in 20 and 30% sucrose solutions for cryoprotection. Then, 50 μm frontal free-floating sections were prepared on a cryotome (Reichert, Vienna, Austria).

To identify neurons expressing tyrosine hydroxylase (TH), an indirect immunohistochemical method was used as described (Efimova et al., 2021). To unmask antigens, the sections were treated with 1% NaBH_4_ for 15 min; to block endogenous peroxidase activity—in 0.3% H_2_O_2_ for 30 min; and to eliminate a non-specific reaction—in 5% normal goat serum (NGS, Vector Labs, Burlingame, CA, United States) in PBS for 90 min. Next, sections were incubated in primary polyclonal anti-rabbit antibodies to TH (Santa Cruz Biotechnology, Cat # sc-14007, RRID: AB_671397, United States, dilution 1:6000) for 70 h at + 4°C, and thereafter, in biotinylated goat anti-rabbit IgG (Vector Labs, Burlingame, CA, United States, catalog number BA-1000, RRID: AB_2313606, United Kingdom, dilution 1:600) for 24 h at + 4°C. Incubation in antibodies was carried out with the addition of 5% NGS and 0.1% NaN_3_. After that, sections were incubated with avidin-biotin horseradish peroxidase complex (ABC Elite system, Vector Labs, Burlingame, CA, United States) for 1 h and processed for a mixture of diaminobenzidine (DAB), NaCl, and 0.03% H_2_O_2_ (Vectastain DAB kit, Vector Labs, Burlingame, CA, United States). Between the above procedures, the sections were washed in 0.01 M PBS.

After the immunohistochemical reaction, the sections were subjected to dehydration in alcohols of ascending concentration, followed by clearing in xylene. Then, the sections were mounted at the glasses and cover slipped in Bio Mount (Bio-Optica, Milan, Italy).

Sections were digitated on an Olympus CX31 microscope (Olympus Corporation, Tokyo, Japan, 10x objective) with a built-in Nikon camera (D3200, Nikon Corporation, Tokyo, Japan). The resulting series of images were processed in Adobe Photoshop 21.1.3. Immunopositive neurons in the ventral tegmental area (VTA) and pars compacta (SNc) and pars reticulata (SNr) of substantia nigra (SN) were manually counted using Fiji ImageJ software (Schindelin et al., 2012). The structures of SN were analyzed at the mediolateral (ML) 1.4 mm, anteroposterior (AP) −3.1 mm, dorsoventral (DV) 4.5 mm level for SNr, and ML 1.1 mm, AP −3.1 mm, DV 4.4 mm level for SNc.

### Adult Neurogenesis Analysis Immunohistochemistry

Brain samples from WT and KO animals (*n* = 5 for each group) were fixed in acetic acid-zinc chloride fixative solution (Naish et al., 1989) and embedded in paraffin using standard methodology. Serial frontal sections, 5 μm in thickness, were prepared on a microtome Microm HM 325 (Thermo Scientific, United States) rotary microtome at several levels: approximately 0.14 mm before the Bregma for the subventricular zone (SVZ) and 1.46 mm behind Bregma for subgranular zone (SGZ) of the dentate gyrus (DG). Immunohistochemical staining with rabbit polyclonal antibodies against Doublecortin (DCX, dilution 1:1,000, Catalog# ab18723, Abcam, United Kingdom) was performed to detect precursor cells (Korzhevskii et al., 2009), while rabbit monoclonal antibodies against Proliferating cell nuclear antigen (PCNA, dilution 1:9,000, Catalog# ab92552, Abcam, United Kingdom) was performed to detect proliferating cells on tissue sections. To unmask antigens, sections were processed in target retrieval solution, modified citrate pH 6.1 buffer (S1700, Agilent, United States) for 22 min and endogenous peroxidase activity was blocked by incubating the sections in 0.3% H_2_O_2_ for 10 min. Non-specific staining was blocked by incubation in Protein Block (Spring Bioscience, United States) for 10 min. Incubation in primary antibodies was performed overnight at 27.5°C. Goat anti-rabbit Horseradish Peroxidase (HRP) Conjugate from Mouse and Rabbit Specific HRP/diaminobenzidine (DAB) immunohistochemistry (IHC) Detection Kit (ab236466, Abcam, United Kingdom) was used as secondary antibodies, and incubation was performed for 25 min at 27.5°C. HRP was visualized using DAB solution (Thermo Scientific, United States). Mounted slides (Cytoseal 60; Richard-Allan Scientific, United States) were examined on the Leica DM 750 light microscope. DCX+ and PCNA+ cells were manually counted in 10 fields of view (with area = 0.503 μm^2^) within the DG and the SVZ (10 ± 1 sections per animal). These values were then recalculated on the 1 mm^2^ area.

### Statistical Analysis

The results of the experiments are expressed as mean ± SEM. Most of our data involved comparison between two groups. Therefore, we first tested data of each parameter for normality using the Shapiro-Wilk test. Due to the normal distribution of these data, a *t*-test with Welsh’s correction was chosen to compare differences between the two groups. In the analysis of locomotor activity for 120 min, as the data contains repeated measures, a two-way repeated measures ANOVA test was performed. For electrophysiological experiments, the data were analyzed using two-way ANOVA. Sidak’s multiple comparisons test was performed to distinguish the most prominent differences.

All statistical analyses were performed by using GraphPad Prism 8 (GraphPad Software, Inc., San Diego, CA, United States). *p* < 0.05 was considered to be statistically significant.

## Results

### Beta-Galactosidase Histochemistry

The insertion of the LacZ reporter gene into the TAAR2 gene made it possible to map the pattern of TAAR2 expression in the mouse brain. The gene LacZ encodes the bacterial enzyme beta-Galactosidase, the activity of which can be visualized (detected) using histochemistry methods. Thus, when beta-Galactosidase interacts with the substrate X-gal (5-bromo-4-chloro-3-indolyl-β-D-galactopyranoside), a blue reaction product is precipitated in cells with beta-Galactosidase (Trifonov et al., 2016). Frontal and sagittal sections of the mouse brain were analyzed.

**FIGURE 2:**
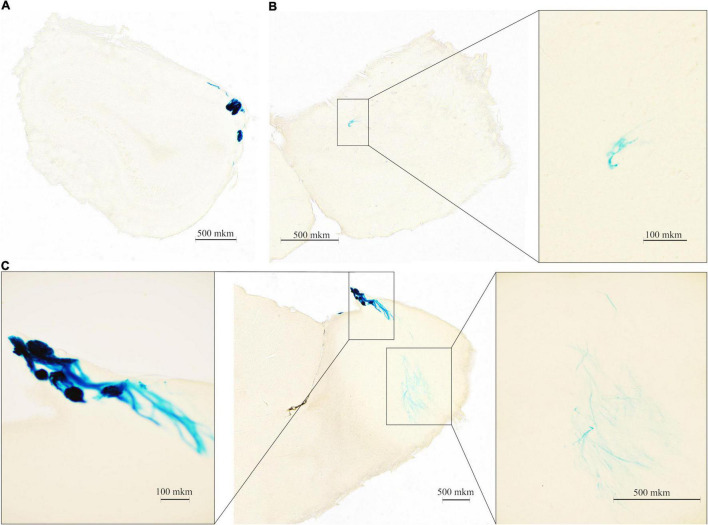
∣ Beta-galactosidase (LacZ) staining shows TAAR2 expression in the mouse olfactory bulb (OB) presented in the frontal and sagittal sections. TAAR2 expression was observed in the glomerular (**A,C** left) and inner (**B,C** right) layers of the OB. Scale bar—500 μm (**A–C,C** right); 100 μm (**B** right, **C** left).

**FIGURE 3:**
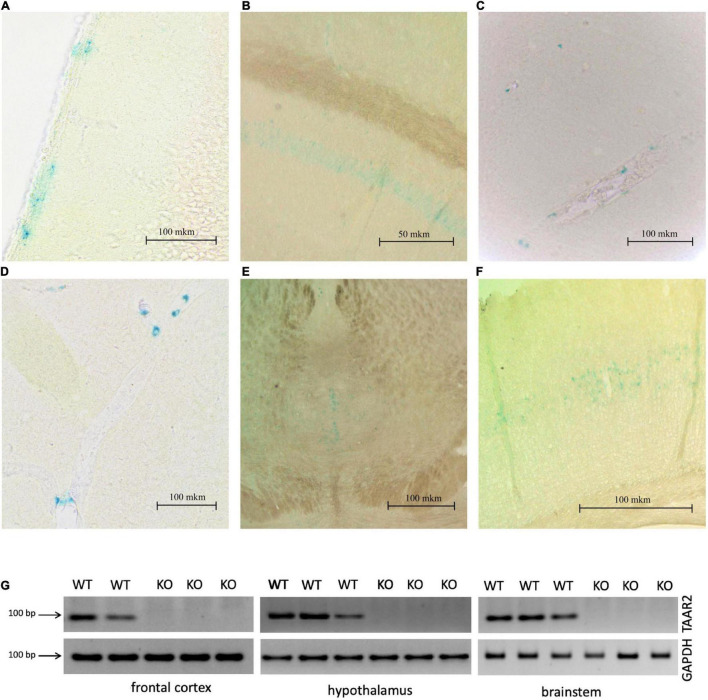
∣ LacZ staining shows TAAR2 expression in the mouse piriform cortex **(A)**, hippocampus (CA1 layer) **(B)**, hypothalamus **(C)**, lateral habenula **(D)**, raphe nucleus **(E)**, somatosensory cortex, layer 5 **(F)**, Scale bar—50 μm **(B)**; 100 μm **(A,C,D–F)**. **(G)** mRNA expression level of TAAR2 in wild type and TAAR-2 KO in frontal cortex, hypothalamus, and brain stem.

It is known that TAARs are expressed in the olfactory bulbs, mainly in the glomeruli of the dorsal part (Pacifico et al., 2012; Dewan, 2021). In agreement, the histochemical reaction was found in the glomerular layer of the olfactory bulb, but intensive staining was found in the deeper layer as well. The histochemical reaction was observed in the fibers of the olfactory nerve, in the glomeruli of the glomerular layer (Figures 2A,C left) several short axon (SA) cells (outer plexiform layer or granular layer) (Figures 2B,C right), and neuronal projections (Figure 2C right) that were visualized throughout the depth of the olfactory bulb. Furthermore, LacZ staining was observed in the limbic areas of the brain receiving olfactory input, i.e., piriform cortex molecular area (Figure 3A) hippocampus (CA1 field, pyramidal layer) (Figure 3B), hypothalamic lateral zone (zone incerta) (Figure 3C), and lateral habenula (Figure 3D). In addition, a histochemical reaction was found in the midbrain raphe nuclei (Figure 3E) and primary somatosensory area of the cortex, layer 5 (Figure 3F). In WT animals, we did not observe any non-specific staining in these areas (data not shown). Real-time quantitative PCR with reverse transcription confirmed TAAR2 gene expression in the mouse brain areas such as the frontal cortex, hypothalamus, and brainstem (Figure 3G). The specificity of designed primers was verified as previously described (Kalinina et al., 2021). Specific bands were detected in all samples from WT, but not TAAR2-KO animals.

### Behavioral Profile

Trace amine-associated receptor 2 knockout mice showed no general health, development, growth, and body weight alterations. In addition, genotype and sex distribution were normal.

**FIGURE 4:**
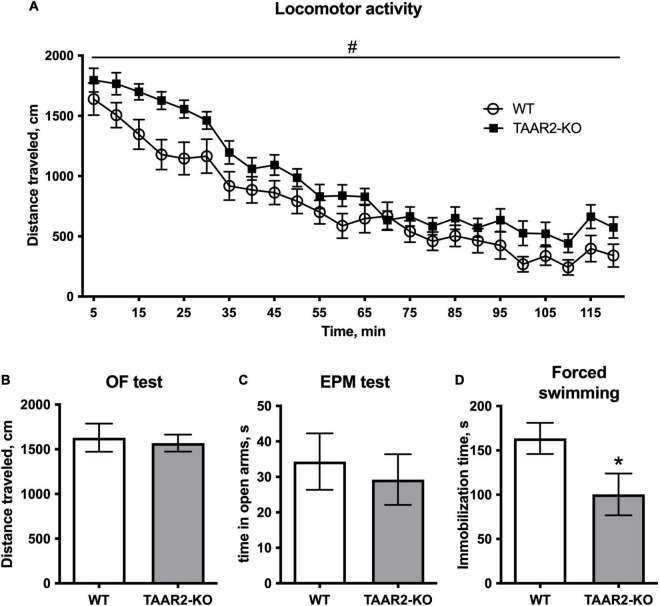
∣ The behavioral parameters of TAAR2-KO vs. wild-type (WT) mice. **(A)** Locomotor activity for 2 h, distance traveled, cm; **(B)** Distance traveled in open field test for 5 min, cm; **(C)** Time spent in open arms in the elevated plus-maze test; **(D)** Immobilization time in the forced swimming test. All data presented as mean ± SEM. *p < 0.05, *t*-test. #*p* < 0.05, two-way ANOVA.

Trace amine-associated receptor 2 knockout mice showed significantly higher horizontal activity in the locomotor activity test during 120 min [*F*(1,34) = 5.843, estimated *p*-value = 0.021] (Figure 4A). Since first 5-min activity of animals in this test serve as a measure of explorative behavior, separate analysis was performed on this time interval that revealed no significant differences between TAAR2-KO and WT mice (*p* = 0.35). Similarly, in the circular open field test, TAAR2-KO mice showed no increase in distance traveled during 5 min of exploration, indicating normal exploratory behavior (Figure 4B). Other parameters, including time in the central zone, number of rearings, and time of grooming were also not different in TAAR2-KO mice compared to WT (Supplementary Figure 1). In the elevated plus-maze test, no difference was found in time spent in open arms (Figure 4C). Distance traveled, time in closed arms, and number of rearings also remained unaltered in TAAR2-KO mice compared to WT mice (Supplementary Figure 1).

In the forced swim test, TAAR2-KO animals had significantly lower immobilization time (100.4 ± 23.59 in the TAAR2-KO group; 163.5 ± 17.64 in the WT group, *p* = 0.046) (Figure 4D). In other behavioral tests, including the social interaction test, light-dark box, marble burying test, and hot plate test, the TAAR2-KO mice showed no differences from WT controls (Supplementary Figure 1).

### Power Spectra of Trace Amine-Associated Receptor 2 Knock-Out vs. Wild-Type Mice

**FIGURE 5:**
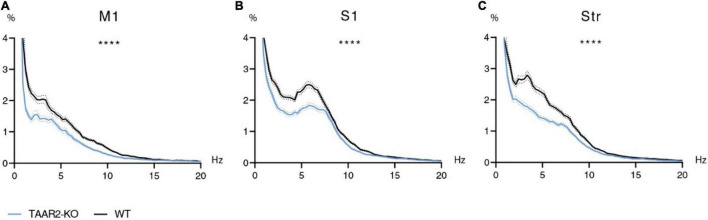
∣ Electrophysiological power spectra in the brain regions of TAAR2-KO vs. WT mice. **(A)** Power spectral density of primary motor cortex (M1) electrocorticogram (ECG). **(B)** Power spectral density of somatosensory cortex (S1) electrocorticogram. **(C)** Power spectral density of local field potentials in the striatum (Str). X-axis—signal frequency in Hz; Y-axis—power spectral density in % of total density. Blue and black lines—power spectra of TAAR2-KO and WT mice, respectively; dotted lines—standard error of the mean (SEM). ****p < 0.0001 two-way ANOVA with Sidak *post hoc* test.

Motor and somatosensory cortex ECG and striatal LFP were recorded in awake and freely moving TAAR2-KO and WT mice, and then analyzed using the Fourier transform. Using two-way ANOVA, power spectral density of the 0.9–20 Hz range was compared. Analysis showed significant differences (*p* < 0.0001) in all three structures (motor cortex, somatosensory cortex, and striatum). The signal’s power spectral density recorded in TAAR2-KO mice was consistently reduced in all studied frequency ranges compared to WT animals (Figures 5A–C). Although the graphs show varying patterns for different structures, the mean spectral power density is higher in WT than in TAAR2-KO animals in all three regions for all studied rhythms (*p* < 0.0001) in all comparisons as follows: *F* values for delta, theta, alpha, and beta ranges, respectively: M1: *F*(1,456) = 37,01; *F*(1,532) = 39,75; *F*(1,456) = 69,17; *F*(1,1064) = 21,56. Str: *F*(1,479) = 50,20; *F*(1,560) = 102,9; *F*(1,480) = 27,05; *F*(1,1099) = 57,34. S1: *F*(1,480) = 30,31; *F*(1,560) = 37,38; *F*(1,480) = 24,44; *F*(1,1119) = 17,73.

### High-Performance Liquid Chromatography Study of Brain Monoamine Levels

**FIGURE 6:**
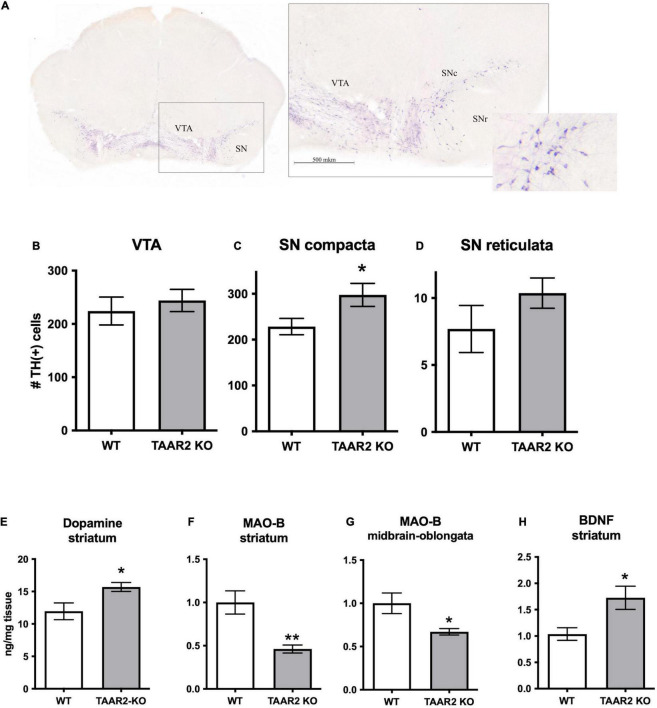
∣ Changes in the nigrostriatal dopamine system of TAAR2-KO mice. **(A)** Distribution of the Tyrosine Hydroxylase (TH) immunopositive (TH+) neurons in the ventral tegmental area (VTA) and the substantia nigra (SN) in TAAR2-KO mice. Countered frontal slice of the mesencephalon with TH-labeled neurons (left) and a microscopic view of an enlarged region marked by the black dashed square (right). SNr, SNc—Substantia Nigra pars reticulata, pars compacta. Scale bar—500 μm. **(B–D)** The number of TH+ neurons in the VTA **(B)**, the Substantia Nigra pars compacta **(C)**, and the Substantia Nigra pars reticulata **(D)**; **(E)** level of dopamine in the striatum tissue ng/mg of tissue; **(F,G)** Monoamine Oxidase (MAO-B) mRNA level in the striatum and midbrain-oblongata; **(H)** mRNA level of brain-derived neurotrophic factor (BDNF) in the striatum. All data presented as mean ± SEM. *p < 0.05; **p < 0.01 *t*-test.

Levels of NE, DA, 5-HT, and metabolites were measured in the frontal cortex, striatum, hypothalamus, and hippocampus in mutant and control mice. The level of DA in the striatum was higher in the TAAR2-KO group compared to WT animals (15.7 ± 0.069 and 11.96 ± 1.292 ng/mg tissue, *p* = 0.025) (Figure 6E). Hippocampal levels of NE were decreased in TAAR2-KO animals in comparison with controls (NE: 0.368 ± 0.013 and 0.442 ± 0.014 ng/mg tissue, *p* = 0.014). All the other parameters measured were not altered in mutant mice (Supplementary Figures 2–6).

### mRNA Expression Level of Key Neuronal Markers

Using real-time PCR (RT-PCR) measurements, we analyzed the expression of common neuronal markers in different brain areas. We showed that TAAR2-KO mice have a decreased level of expression of Monoamine Oxidase B (MAO-B), the enzyme that catalyzes the oxidative deamination of biogenic amines (in particular, trace amine β-phenylethylamine) in the striatum (*p* = 0.009) and midbrain (*p* = 0.039) (Figures 6F,G). Furthermore, TAAR2-KO mice had an increased the brain-derived neurotrophic factor (BDNF) level in the striatum (Figure 6H) (*p* = 0.026). For other studied mRNA levels, we have not found significant differences between TAAR2-KO and WT animals (Supplementary Figures 7–9).

### Tyrosine Hydroxylase Immunohistochemistry

To quantitatively evaluate the status of the DA system, DA cell bodies containing brain structures (VTA, SNc, SNr) of WT and TAAR2-KO mice were assessed for TH-immunopositive (TH+) neurons. The border between the SN and VTA was identified with light-colored reticular tissue passing between the structures. Immunopositive neurons were characterized by a dark stained predominantly oval-shaped soma and rare dendrites (Figure 6A). A significantly higher number of the TH+ neurons was detected in TAAR2-KO mice in the SNc (228.6 ± 17.80 and 297.5 ± 25.08 per slice for WT and TAAR2-KO mice, respectively, *p* = 0.037) (Figure 6B), but no significant differences were observed in the SNr (7.7 ± 1.76 and 10.36 ± 1.13 per slice for WT and TAAR2-KO mice, respectively, *p* = 0.22) and the VTA (224.2 ± 26.18 and 244.1 ± 20.86 per slice for WT and TAAR2-KO mice, respectively, *p* = 0.6) (Figures 6C,D).

### Adult Neurogenesis Analysis

**FIGURE 7:**
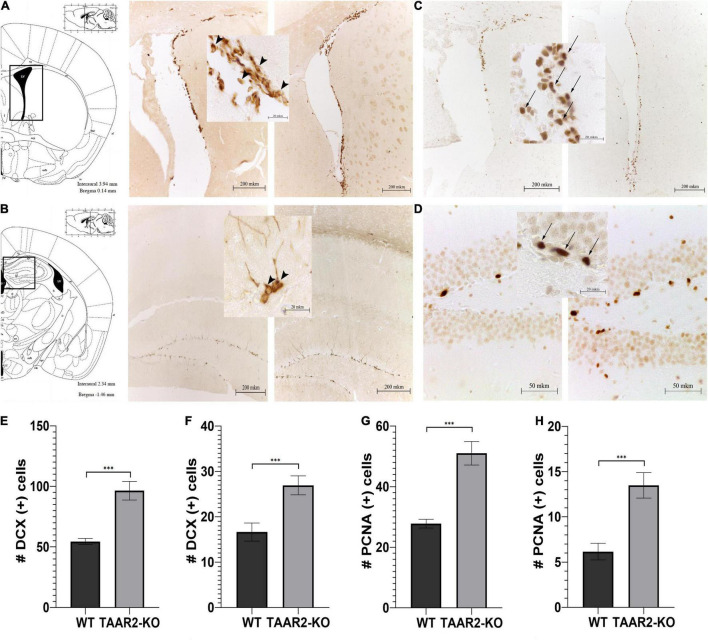
∣ TAAR2-KO mice have an increased number of neuroblast-like and proliferating cells in subventricular (SVZ) and subgranular (SGZ) zones in comparison to WT animals. **(A,B)** Representative micrographs of doublecortin (DCX)-positive cells in mentioned brain structures of WT (left) and TAAR2-KO (right) mice; **(C,D)** representative micrographs of proliferating cell nuclear antigen (PCNA)-positive cells in mentioned brain structures of WT (left) and TAAR2-KO (right) mice; **(E,F)** the number of DCX-positive cells in SVZ and SGZ of WT and TAAR2-KO mice; **(G,H)** number of PCNA-positive cells in SVZ and SGZ of WT and TAAR2-KO mice. *n* = 5; ***p < 0.001 by *t*-test. Arrowheads indicate DCX-positive cells, while arrows indicate PCNA-positive cells.

The immunohistochemistry reactions against DCX and PCNA revealed neuroblast-like DCX+ cells and proliferating PCNA+ cells, respectively, in the SVZ and the SGZ. It has been found that the number of DCX+ cells significantly increased in both SVZ and SGZ of TAAR2-KO mice in comparison to WT animals (*p* < 0.001) (Figures 7E,F). Interestingly, in TAAR2-KO but not WT mice, we detected not only cells entering their canonical migratory route through the rostral migratory stream (RMS) from SVZ (Figures 7A,C) but also a certain amount of migrating DCX+ cells in the white matter above the hippocampus (Figure 7B). Since their migratory pathway does not match RMS, future studies would be necessary to determine where these cells migrate. The number of proliferating PCNA+ cells in SGZ and SVZ (Figures 7C,D) was markedly lower than DCX+ cells, but the cell counts were significantly higher in TAAR2-KO mice compared to control mice in both regions (*p* < 0.001) (Figures 7E–H). Notably, some PCNA+ and DCX+ cells were found not only in the SVZ but also inside the wall of the lateral ventricles (as seen in Figures 7A,C).

## Discussion

Soon after discovering TAAR receptors (Borowsky et al., 2001; Bunzow et al., 2001), pharmacological research has focused mainly on the TAAR1 receptor (Berry et al., 2017) as it was considered the only one present in the brain (Liberles and Buck, 2006). However, accumulating evidence indicates that the TAAR1 receptor is not the only member of the TAAR family involved in brain functions. In addition, other TAAR receptors could also be a point of interest for neuropharmacology. For example, the previously considered “olfactory” TAAR5, and now, in TAAR2, receptors are found in the brain and contribute to regulating certain brain functions (Espinoza et al., 2020; Efimova et al., 2021). While the expression of TAAR5 in specific brain structures was reported earlier, the expression of TAAR2 in the brain has not been demonstrated previously (Gainetdinov et al., 2018). Here, we used LacZ expressing TAAR2-KO mice to show localization of TAAR2 in several brain areas. Previously, the expression of TAAR2 was shown only in the olfactory epithelium and the glomerular layer of the olfactory bulb (Pacifico et al., 2012; Dewan, 2021). We confirmed that TAAR2 receptors can not only be found in the olfactory epithelium and the glomeruli of the olfactory bulb, but also in deeper layers of the olfactory bulb (mitral/tufted cells), projecting to the limbic brain areas. Furthermore, we observed the expression of TAAR2 in several brain regions mostly related to the limbic system—the piriform cortex, the hippocampus, the habenula, the hypothalamus, and in the raphe nuclei and the somatosensory cortex. The expression of mouse TAAR1 (Lindemann et al., 2008; Berry et al., 2017) and TAAR5 (Espinoza et al., 2020) receptors has also been shown primarily in limbic and related structures. However, each member of the TAAR family seem to have a distinct expression pattern. With regard to mouse TAAR5 LacZ expression data, a concern was raised that the gene targeted allele used to report TAAR5 expression retained a neomycin resistance gene selection cassette, which might cause the abnormal gene expression in mTaar5 (Yoon et al., 2015; Dewan, 2021). Importantly, TAAR2-KO mice, in which a neomycin resistance gene selection cassette was removed through CREed deletion, had similar to TAAR5 predominantly limbic pattern of LacZ expression. Furthermore, recent transcriptomic analyses of TAAR5 expression in the human brain also showed ubiquitous low TAAR5 expression in the cortical and limbic brain areas, the amygdala and the hippocampus, the nucleus accumbens, the thalamus, the hypothalamus, and others (Vaganova et al., 2021). A similar conclusion was independently achieved in another human transcriptomic analysis study where predominantly limbic expression of all TAARs, including TAAR2, was reported with TAAR5 appearing to have the highest expression level (Gaudel et al., 2021).

Trace amine-associated receptor 2, like TAAR1 and TAAR5 receptors, exerted modulatory action on the brain monoamine systems (Leo et al., 2014; Espinoza et al., 2020). Despite being expressed in the raphe nuclei where cell bodies of serotonin neurons are localized, the lack of TAAR2 receptors had a limited effect on the serotonin system as evidenced by the unaltered brain 5-HT and 5-HIAA levels in mutant mice. As regards the dopamine system, TAAR2 receptors seem to have a greater role since, in TAAR2-KO mice, the striatal levels of dopamine were elevated. Elevated dopamine levels in TAAR2-KO mice could be due to several reasons. Potentially, it can be explained by a decrease in the mRNA level of MAO-B, one of the dopamine metabolizing enzymes, observed in the striatum and brainstem of TAAR2-KO mice. However, it should be noted that the lack of MAO-B in mice did not affect striatal DA levels, arguing against such a possibility (Shih et al., 1999). Therefore, it is more likely that the elevation of dopamine levels in the striatal tissue could be related to increased dopaminergic neuron number in the SNc of TAAR2-KO mice.

Elevated striatal dopamine in TAAR2-KO mice correlates well with the observed behavioral changes. Dopamine is a critical neurotransmitter regulating locomotor activity, and elevation of dopamine level results in hyperlocomotion (Molinoff and Axelrod, 1971; Gainetdinov et al., 1999). Therefore, increased locomotor activity in TAAR2-KO mice could result from elevated striatal dopamine. The increased locomotor activity may also contribute to the decreased immobilization in the forced swim test, which is usually considered a marker of depression-like behavior (Porsolt et al., 1977). Further studies are necessary to determine if a decrease in immobilization could indicate the contribution of TAAR2 to emotional behaviors or be a result of the increase of the general activity of TAAR2-KO mice. At the same time, we noted no alterations in anxiety-related behaviors of mutants that correspond well with the lack of alterations in the brain serotonin system.

We observed significant alterations in ECoG in the motor cortex and LFP in somatosensory cortex and striatum of TAAR2-KO mice compared to WT animals. Power spectra demonstrated a significant decrease in power density in all three studied structures, which allows us to assume that TAAR2 plays a part in regulating brain oscillations of a wide frequency range (0.9–20 Hz). Electroencephalogram (EEG) power alterations have been reported in a number of neuropsychiatric disorders (Han et al., 2013; Tsolaki et al., 2014; Newson and Thiagarajan, 2019). EEG/ECoG alterations have also been observed in pharmacological and transgenic rodent models of brain disorders (Drinkenburg et al., 2016; Kent et al., 2018). In most of these cases, studies report increases in specific frequency ranges. However, decreases in EEG power of specific rhythms have been reported for some human disorders (PTSD, autism, ADHD) and noted in mouse models of Alzheimer’s disease (Kent et al., 2018; Newson and Thiagarajan, 2019). Specific monoaminergic changes may also lead to EEG/LFP power changes. In our case, a decrease in delta oscillations in the striatum may be related to increased dopamine levels (Whalen et al., 2020). Notwithstanding all the causes mentioned above for EEG changes in humans and rodents, a significant power decrease on such a wide frequency range (0.9–20 Hz) as we observed in TAAR2-KO mice is rare. A similar effect has been found in healthy human aging as spectral power of all EEG rhythms decreases with age to different degrees (Vysata et al., 2012; Vlahou et al., 2014). At this point, we are unable to determine any correlations or causes for such an effect in TAAR2-KO mice. Yet, a robust difference like this allows us to assume that lack of TAAR2 affects brain activity in specific regions, be it directly or indirectly. It should be noted that electrophysiological features of TAAR2-KO mice proved to be different from those observed in TAAR5-KO mice, which have been shown previously (Kalinina et al., 2021).

The increased number of dopamine neurons in TAAR2-KO mice is a matter of particular interest. It correlated well with elevated striatal levels of BDNF and increased adult neurogenesis markers in major neurogenic zones SVZ and SGZ in mutant mice. A similar pattern was observed previously in TAAR5-KO mice, but instead of BDNF, another growth factor GDNF was increased (Efimova et al., 2021). Neurotrophic factors, such as BDNF and GDNF, are well-known regulators of the dopamine system and are described as candidates for treating Parkinson’s disease as they exhibit dual neuroprotective and neurogenic properties (Lindholm and Saarma, 2021). However, it is unclear whether increased dopamine innervation occurred developmentally or due to increased adult neurogenesis of dopamine neurons, an intriguing possibility remaining a subject of intensive debate (Farzanehfar, 2018; Mourtzi et al., 2021). Therefore, further detailed studies with additional neurogenesis markers, including Bromodeoxyuridine (BrdU), are necessary to determine the cause and mechanism of the increased number of dopamine neurons under the condition of TAAR2 and TAAR5 deficiency.

These observations indicate many TAAR5 knock-out mice changes similar to those observed in TAAR2-KO mice—effects on behavior, monoamine systems, number of dopamine neurons, and adult neurogenesis. Furthermore, these studies show that not only TAAR1, but also other TAARs are present in the brain and are relevant to the regulation of brain functions. Therefore, it seems that all TAARs as a class can have a similar mechanism of action. However, taking into account differences in the expression pattern and effects on behavior, electrophysiological activity, and monoamine neurochemistry, each TAAR subtype should have its own distinct spectra of action. Thus, not only TAAR1, but also TAAR5, TAAR2, and likely other TAAR family members could represent a significant interest for pharmacology. Hence, targeting these receptors may provide a novel multifaceted approach for the treatment of brain disorders. With regard to TAAR2, particularly intriguing would be a further detailed investigation of the mechanism by which TAAR2 can affect dopamine transmission and evaluation of the potential of future TAAR2-based therapies for the treatment of Parkinson’s disease.

## Data Availability Statement

The original contributions presented in the study are included in the article/Supplementary Material, further inquiries can be directed to the corresponding author.

## Ethics Statement

The animal study was reviewed and approved by Bioethics Committee of St. Petersburg State University, St. Petersburg, Russia.

## Author Contributions

EE and RG designed the study, analyzed the data, and wrote the manuscript. SK, MM, NK, TS, VR, MP, AK, RM, DS, AVe, NM, AVo, PM, DK, and EB performed the experiments, analyzed the data, and contributed to the writing of the manuscript. All authors revised and approved the final manuscript.

## Conflict of Interest

The authors declare that the research was conducted in the absence of any commercial or financial relationships that could be construed as a potential conflict of interest.

## Publisher’s Note

All claims expressed in this article are solely those of the authors and do not necessarily represent those of their affiliated organizations, or those of the publisher, the editors and the reviewers. Any product that may be evaluated in this article, or claim that may be made by its manufacturer, is not guaranteed or endorsed by the publisher.
