# Supplementary material for: Trace Amine-Associated Receptor 2 Is Expressed in the Limbic Brain Areas and Is Involved in Dopamine Regulation and Adult Neurogenesis
Source: Front Behav Neurosci. 2022 Apr 1;16:847410. doi: 10.3389/fnbeh.2022.847410 (PMC9011332; doi:10.3389/fnbeh.2022.847410)
Supplement: Supplementary file 1 [file Data_Sheet_1.PDF]

Supplementary material

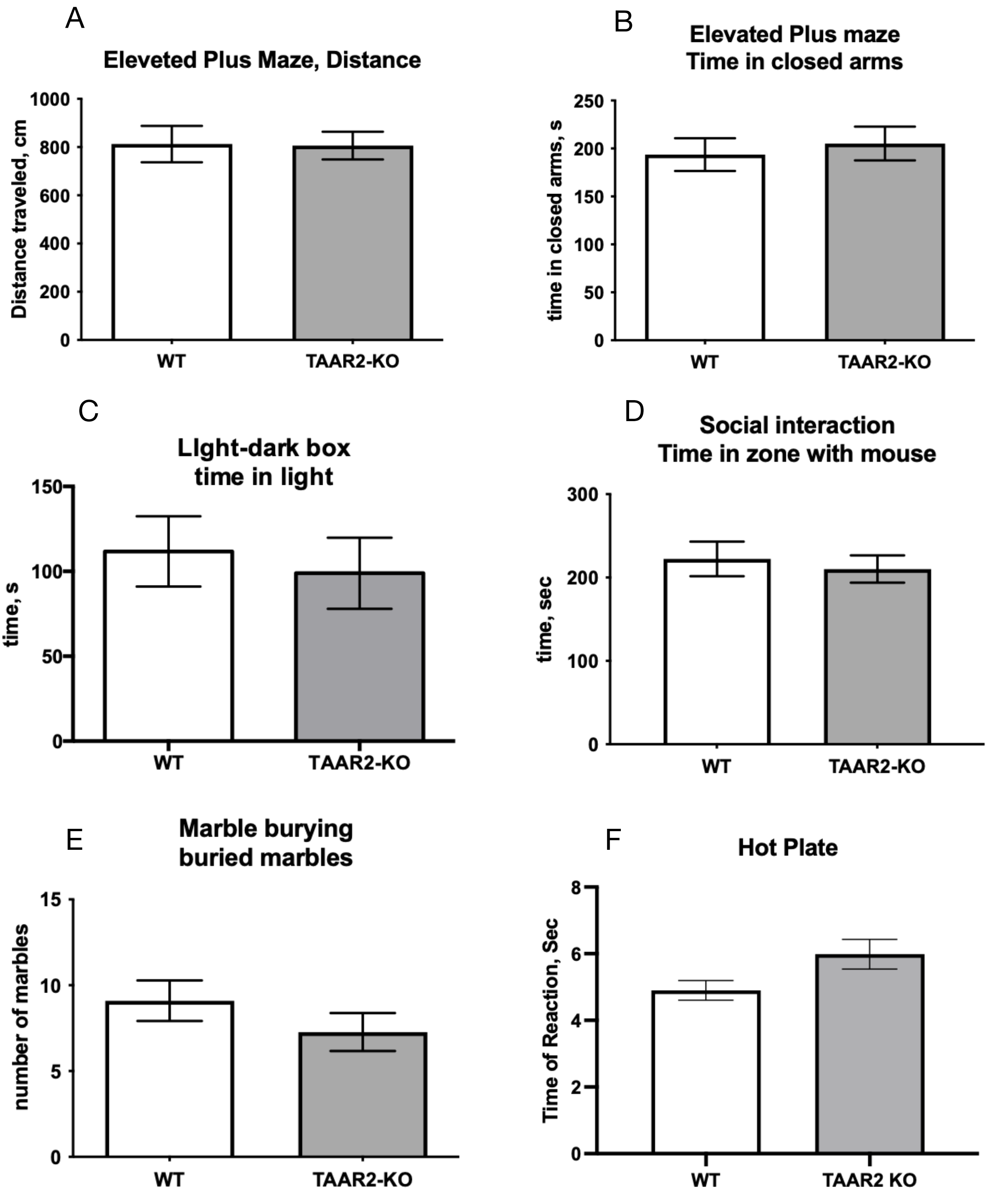

Fig. S1. Behavior of WT and TAAR2-KO mice in (A) Elevated Plus-maze, distance moved, (B) Elevated plus maze, time in closed arms, (C) Light-dark box test, time in light zone (sec); (D) Social interaction test, time in the zone with the unfamiliar mouse; (E) Marble burying test, number of marbles buried; (F) Hot plate test, time of reaction. WT (N=10), TAAR2-KO (N=11). All data presented as mean±SEM.

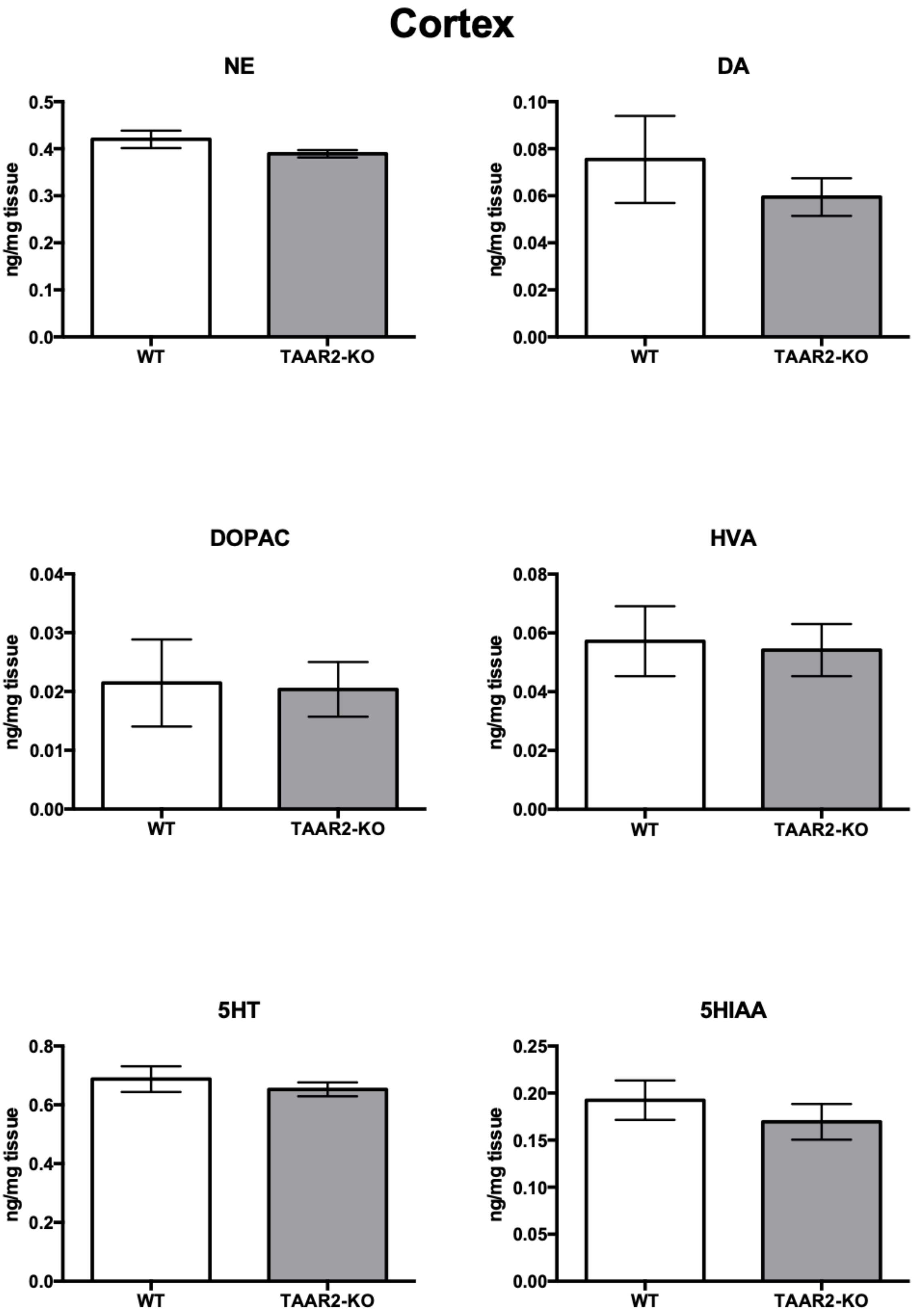

Fig. S2. Tissue content of monoamines and their metabolites in the frontal cortex of WT and TAAR2-KO mice. WT (N=9), TAAR2-KO (N=12). All data presented as mean±SEM.

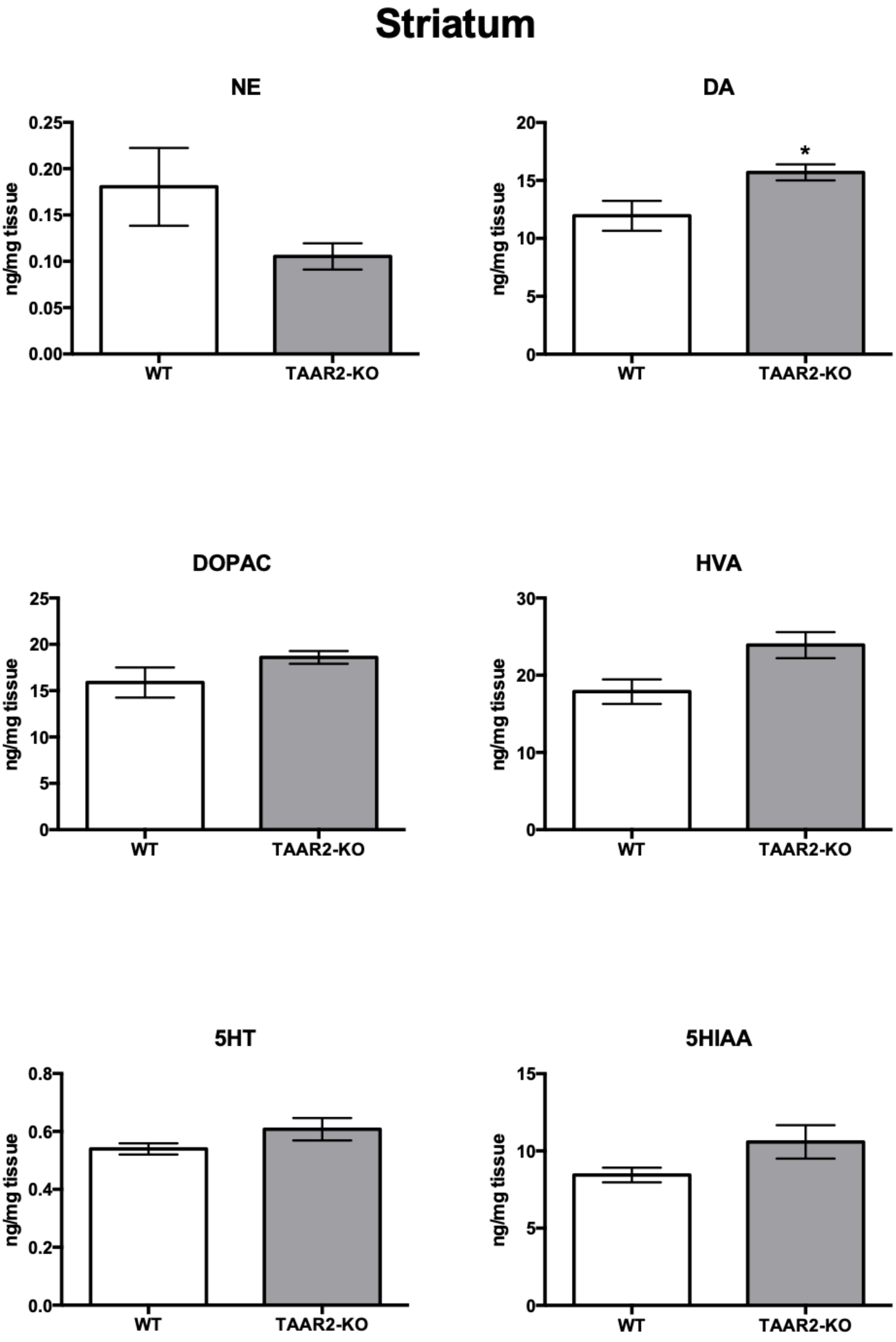

Fig. S3. Tissue content of monoamines and their metabolites in the striatum of WT and TAAR2-KO mice. WT (N=9), TAAR2-KO (N=12). All data presented as mean±SEM. * - $p<0.05$, t-test

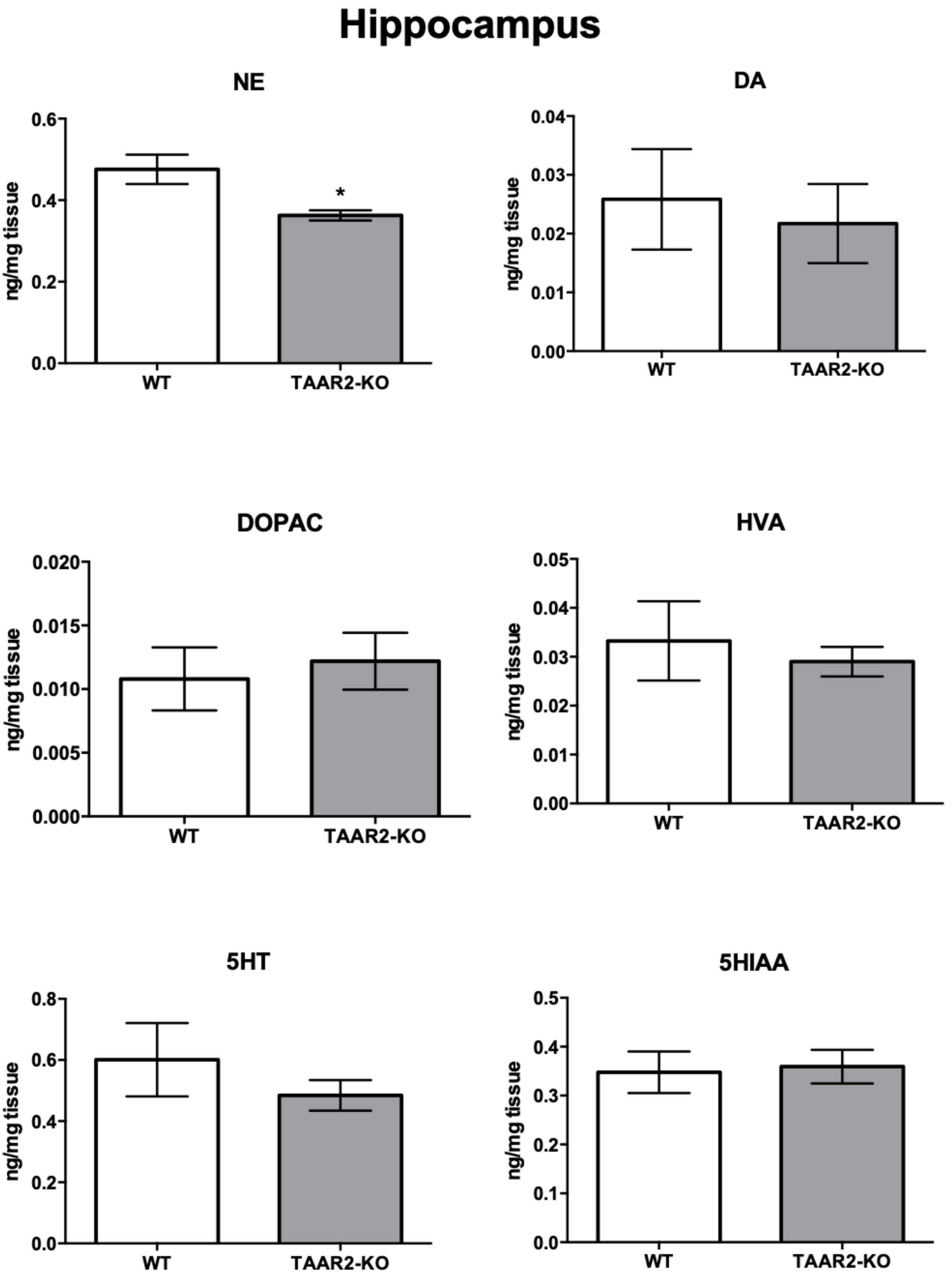

Fig. S4. Tissue content of monoamines and their metabolites in the hippocampus of WT and TAAR2-KO mice. WT (N=9), TAAR2-KO (N=12). All data presented as mean±SEM. * - $p<0.05$, t-test.

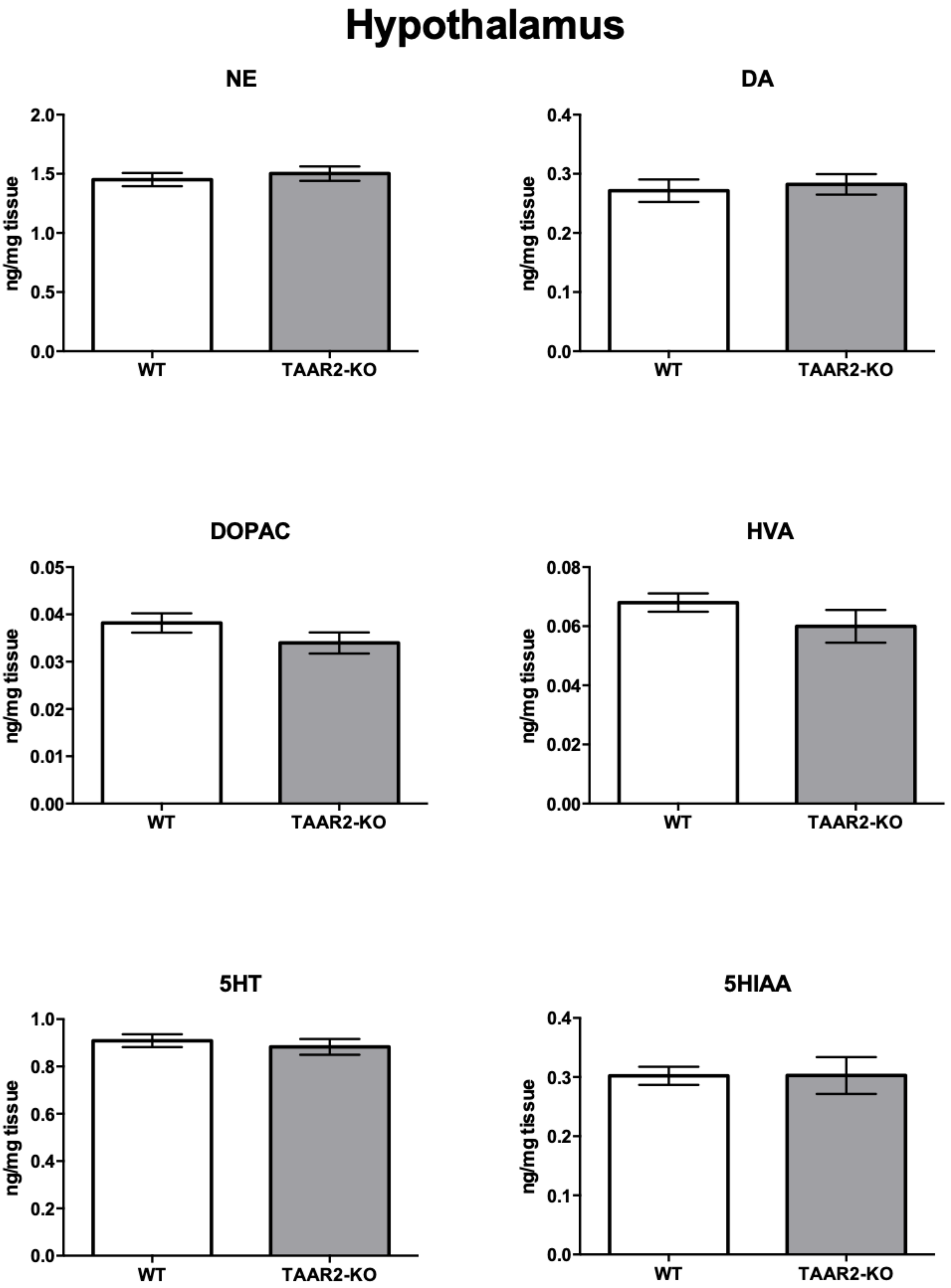

Fig. S5. Tissue content of monoamines and their metabolites in the hypothalamus of WT and TAAR2-KO mice. WT (N=9), TAAR2-KO (N=12). All data presented as mean±SEM

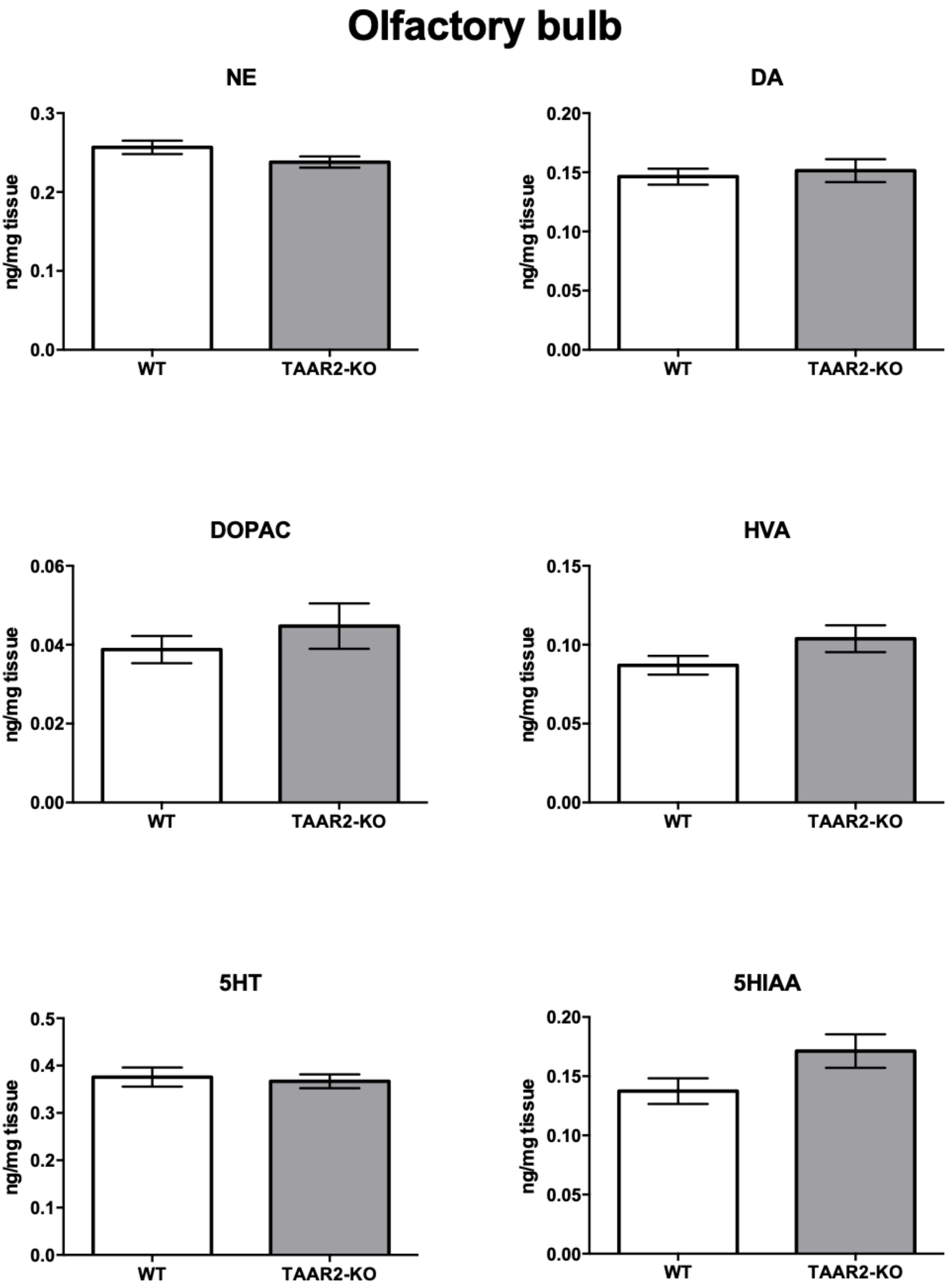

Fig. S6. Tissue content of monoamines and their metabolites in the olfactory bulb of WT and TAAR2-KO mice. WT (N=9), TAAR2-KO (N=12). All data presented as mean±SEM.

Cortex

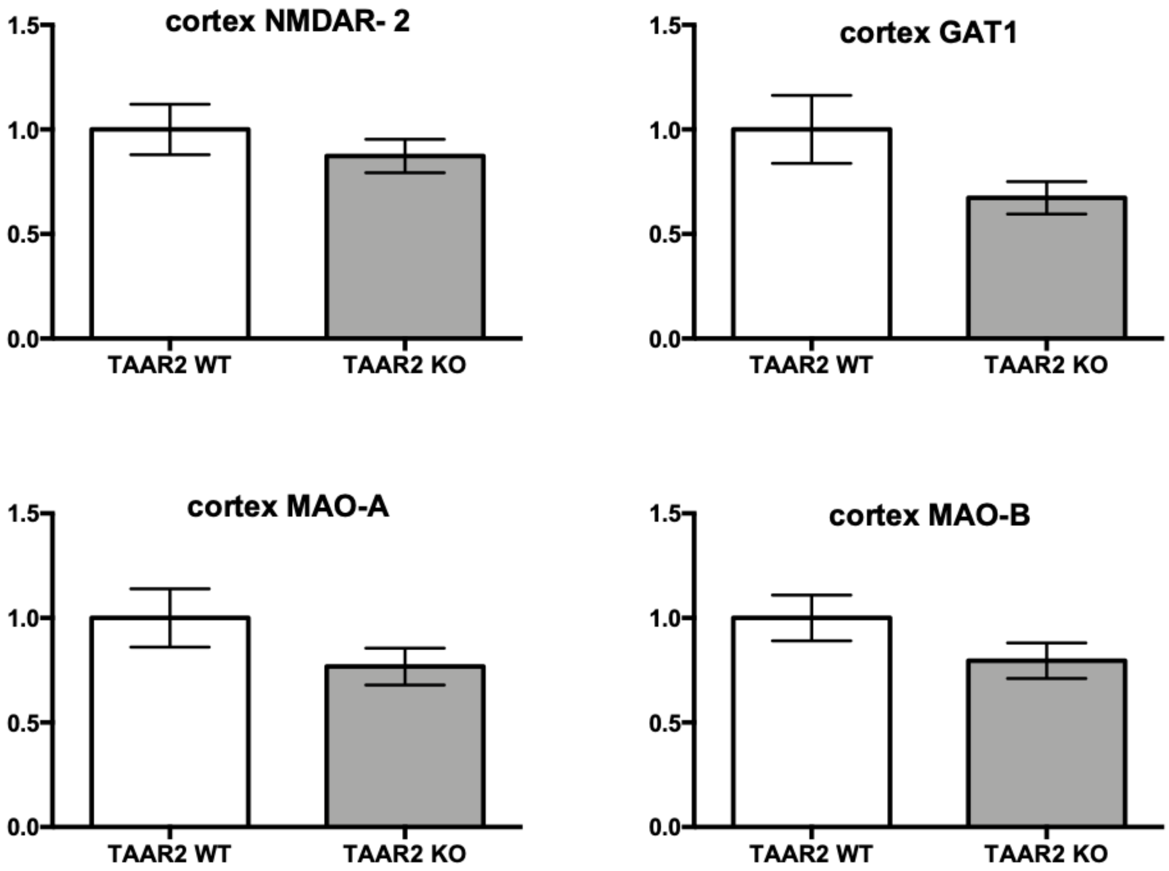

Fig. S7. Expression level of key neuronal markers mRNA in WT and TAAR2-KO mice measured in the cortex tissue. WT (N=6), TAAR2-KO (N=6). All data presented as mean±SEM.

Striatum

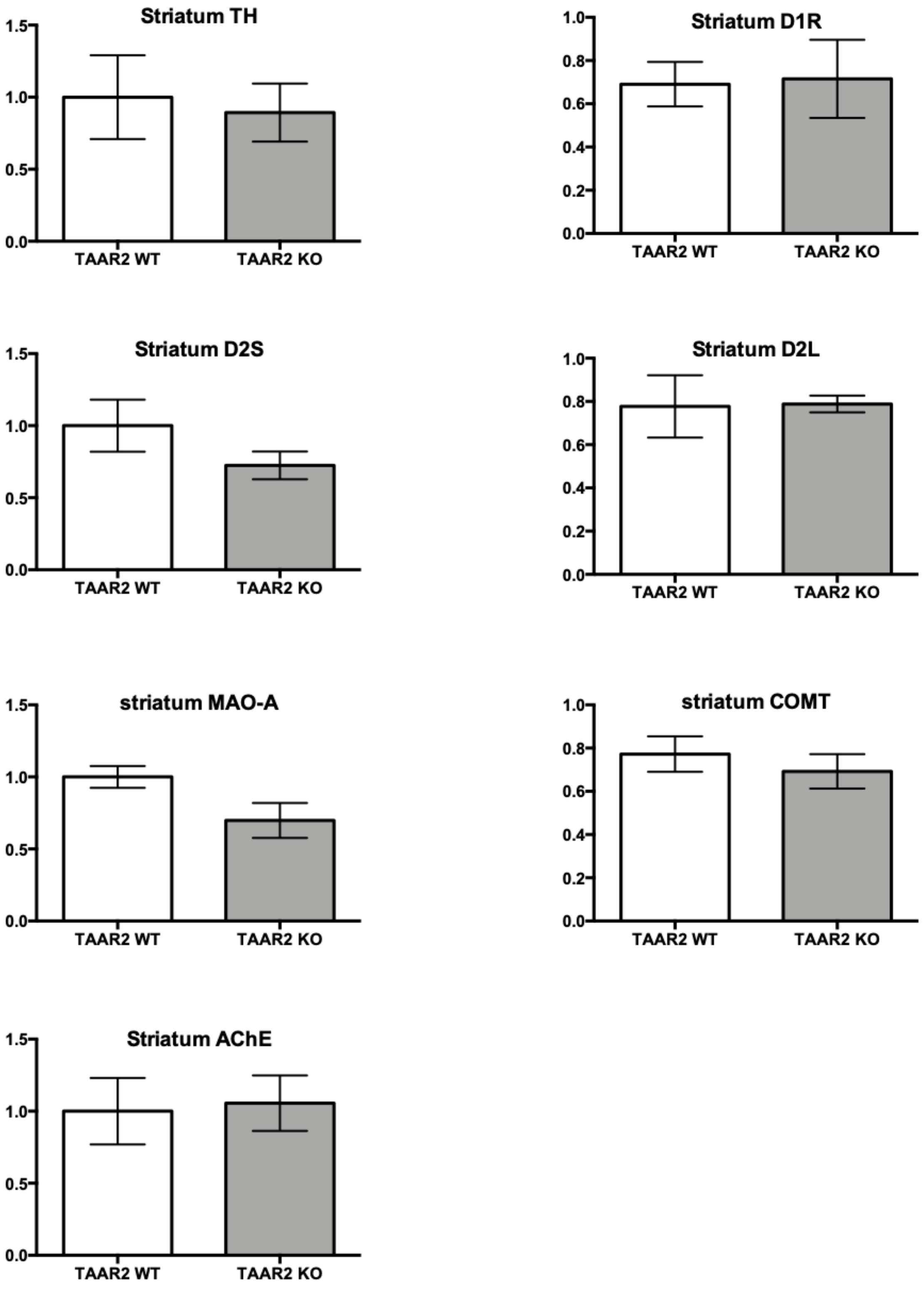

Fig S8. Expression level of key neuronal markers mRNA in WT and TAAR2-KO mice measured in the striatum tissue. All data presented as mean±SEM

Midbrain/medulla oblongata

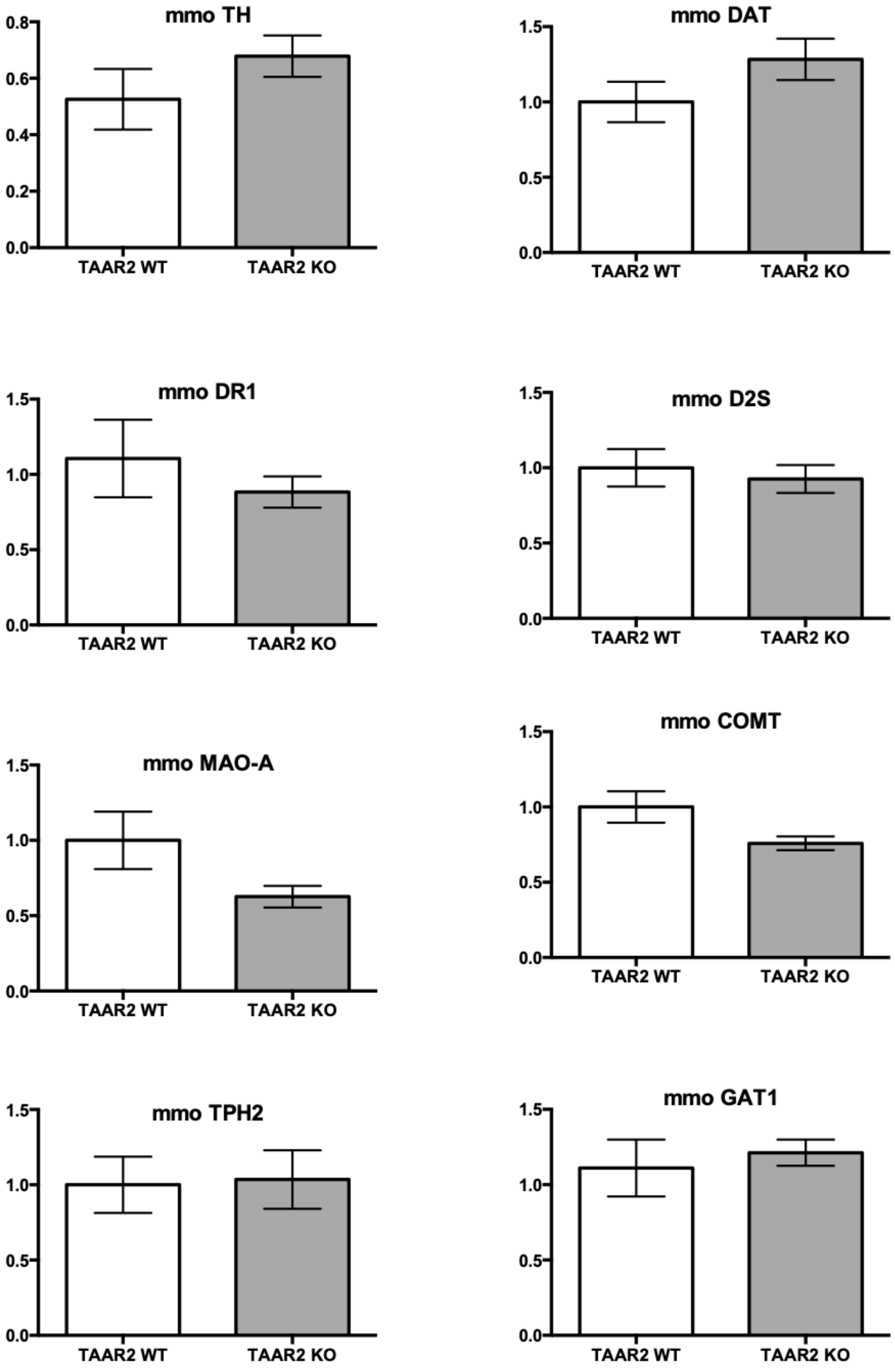

Fig. S9. Expression level of key neuronal markers mRNA in WT and TAAR2-KO mice measured in the midbrain/medulla oblongata tissue. All data presented as mean±SEM.
